# High migratory potential of fall armyworm in West Africa despite stable temperatures and widely available year‐round habitats

**DOI:** 10.1111/1744-7917.13502

**Published:** 2025-01-19

**Authors:** Fan‐Qi Gao, Hui Chen, Rosina Kyerematen, Gao Hu, Regan Early, Jason W. Chapman

**Affiliations:** ^1^ Centre for Ecology and Conservation University of Exeter Penryn Cornwall United Kingdom; ^2^ Department of Biology University of Lund Lund Sweden; ^3^ Department of Entomology Nanjing Agricultural University Nanjing China; ^4^ Department of Animal Biology and Conservation Science University of Ghana Accra Ghana

**Keywords:** China, fall armyworm, flight performance, Ghana, migration, morphological features

## Abstract

The fall armyworm (FAW), an important migratory pest native to the Americas, was first detected in a nonnative region (West Africa) in 2016. In the following years, it quickly spread to multiple regions worldwide. FAW exhibits long‐distance seasonal migration in both the Americas and Asia, primarily to take advantage of suitable seasonal habitats as they appear along the migratory pathways. Tropical West Africa experiences minimal annual temperature variation and has widely distributed potential year‐round habitats, leading us to hypothesize that the migration capacity of FAW populations in this region may be substantially reduced. To test our hypothesis, we assessed the flight performance of FAW collected from Ghana in West Africa with tethered flight mills and compared it to that of a FAW population from southern China. Additionally, we quantified the relationships between morphological characteristics and flight performance of the FAW from Ghana. Based on observed flight behaviors, we categorized FAW into migratory and non‐migratory types. The flight capabilities of first‐generation Ghanaian FAW bred in the laboratory were similar to that of the field population from Yunnan, Southwest China, with migrants making up the majority. However, after several generations of laboratory rearing, the flight capability of the Ghanaian population significantly declined, primarily due to a marked increase in the proportion of non‐migratory individuals. The low correlation between morphological variables and flight duration suggests that genetic factors likely determine most variations in flight propensity. The results of this study indicate that FAW with high migratory capacity in West Africa is likely to pose a threat to crops in eradication zones and neighboring uninvaded areas and may possibly be capable of crossing the Sahara Desert and invading Europe. Therefore, it is crucial to establish comprehensive pest early warning and management systems.

## Graphical abstract

Despite the minimal annual temperature variation and the widespread distribution of potential year‐round habitats in Ghana, the migratory potential of the local fall armyworm remains strong and is indistinguishable from that of the fall armyworm in China, which undergo seasonal migration.

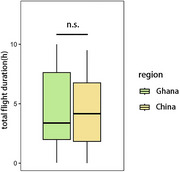

## Introduction

Migration will only evolve when the fitness benefits of migration, on average, outweigh staying in the current habitat (Shaw & Levin, 2011). Many insects are migrants and exhibit migratory polymorphism, with habitat availability influencing their migration decisions (Dingle & Drake, 2007; Roff & Fairbairn, 2007). Deteriorating environments may lead to large‐scale migration, and many insect species reproduce only after migrating. This behavior helps expand species’ reproductive efforts spatially by dispersing offspring across multiple potentially suitable areas (Holland *et al.*, 2006). This strategy of risk‐spreading through migration provides additional benefits to migratory insects compared to most insects that survive through adverse periods through diapause or quiescence, ensuring continuous reproduction of the species throughout the year (Chapman *et al.*, 2012; Chapman *et al.*, 2015). However, migration is costly and risky. Migrants expend energy on developing flight machinery and fuel reserves, potentially compromising immunity, and fecundity. They are also at risk of being blown to unsuitable areas by unfavorable winds, resulting in high mortality (Chapman *et al.*, 2015). Therefore, when the conditions in the current habitat are suitable, it might be more beneficial for insects to allocate energy to reproduction rather than long‐distance flight. For example, studies have shown that in wing‐dimorphic brown planthoppers, the proportion of short‐winged, non‐migratory individuals, which have higher reproductive potential compared to the long‐winged migratory individuals, increases after colonizing a new area with plentiful resources for breeding (Lin *et al.*, 2018).

Initially confined to tropical and subtropical regions of the Americas, the fall armyworm (FAW; *Spodoptera frugiperda*), is a notorious migratory pest with the capability of larval development occurring on >350 host plant species (Montezano *et al.*, 2018), and regularly causing significant damage to major crops such as corn, cotton, rice, and soybeans (Barros *et al.*, 2010; Kenis *et al.*, 2022). It was first observed outside its native range in West Africa in 2016, and within 2 years, it invaded most countries in Sub‐Saharan Africa. FAW was then detected in India, Myanmar, China, and other Asian countries in 2018, subsequently reaching as far as Japan by 2019, Israel and Australia in 2020, and New Zealand by 2022 (Kenis *et al.*, 2022). The chronology of the detection pattern has suggested a west‐to‐east spread, from the Americas into West Africa, then East Africa, India, Southeast Asia and East Asia (Nagoshi *et al.*, 2019; Nagoshi *et al.*, 2020; Kenis *et al.*, 2022; Nagoshi *et al.*, 2022). However, analyses of genomic signatures have recently produced a competing hypothesis, of multiple complex introductions involving east‐to‐west as well as west‐to‐east invasions (Tay *et al.*, 2022; Rane *et al.*, 2023; Tay *et al.*, 2023). Recently, FAW was detected in Turkey during 2022 and in Cyprus during 2023 (EPPO, 2023a, 2023b), and captured in Romania during 2024 (Cean *et al.*, 2024), indicating that FAW may be on the brink of establishing in Europe. The rapid global spread of FAW can lead to economic losses of up to 73% in corn production (Kenis *et al.*, 2022).

In its native range, FAW is distributed year‐round across most of South and Central America, the Caribbean, and the southern regions of Texas and Florida in the United States (Vickery, 1929; Johnson, 1987; Casmuz *et al.*, 2010). In spring, FAW seasonally migrates northward, causing seasonal infestations in most states east of the Rocky Mountains in the United States and even in southern regions of Canada. (Johnson, 1987; Kenis *et al.*, 2022). In the Chinese portion of its nonnative range, FAW also engages in seasonal migrations to adapt to environmental changes. FAW established a seasonal migratory pattern upon invading Asia in its second year. They overwinter in the Indochinese Peninsula and South China and migrate during warmer seasons toward Northeast China, Japan, and the Korean Peninsula (Ma *et al.*, 2019; Chen *et al.*, 2020; Li *et al.*, 2020; Wu *et al.*, 2022). Both the United States and China are situated in the northern hemisphere with a wide range of latitudes. Consequently, regions farther away from the equator in these 2 countries exhibit seasonal abundance in crop resources, with periods of resource scarcity or adverse climatic conditions. FAW utilizes seasonal migration to exploit these temporary food supplies and favorable conditions for survival and reproduction, while ensuring timely retreat when environmental conditions deteriorate (Westbrook *et al.*, 2016).

While seasonal migration is observed in both American and Asian FAW, the migration strategy of African FAW populations remains unclear. Considering climate and environmental conditions present over much of Sub‐Saharan Africa, long‐range north‐south migrations may not be beneficial in many regions, and in some locations may be extremely risky. North Africa features the vast Sahara Desert, where the high temperatures and lack of food resources could lead to the complete demise of FAW populations migrating northward (Wang *et al.*, 2023). Additionally, tropical regions immediately south of the Sahara experience minimal temperature variation throughout the year but have distinct rainy and dry seasons. Suitable habitats are primarily associated with unpredictable rainfall, making regular north‐south migrations unlikely to be beneficial for tracking randomly distributed suitable habitats (Sappington, 2024). This suggests that FAW populations in tropical Africa may have reduced their migratory capacity after arriving in Africa. If this is indeed the case, it indicates that the migration strategy of the FAW may rapidly adjust to adapt to local habitats. Therefore, we propose 2 competing hypotheses: (1) The natural environment of Sub‐Saharan Africa has led to a rapid reduction in migratory behavior in FAW populations in tropical West Africa, and migratory activity will be significantly reduced compared to East Asian populations which have maintained high migratory activity (Chen *et al.*, 2022; Chen *et al.*, 2023). (2) Despite living in year‐round suitable regions for multiple generations, FAW populations in tropical West Africa still maintain the high migratory ability inherited from their American ancestors (Chen *et al.*, 2023).

Certain morphological traits, such as body mass reflecting fuel reserves, and flight apparatus parameters like forewing area, forewing length, wing loading, and wing aspect ratio affect insect migratory performance (Yao & Zhang, 2001; Minter *et al.*, 2018; Freedman *et al.*, 2020; Jyothi *et al.*, 2021). We aim to investigate whether the morphological characteristics of FAW play a decisive role in its migratory performance through this experiment.

To test the above hypotheses regarding whether the migratory capacity of FAW populations from tropical Africa have decreased, we measured and compared the flight behavior of FAW populations collected in West Africa (Ghana) and Southwest China (Yunnan). Additionally, we measured morphological parameters of the Ghanaian population and correlated them with flight performance to explore potential associations between migratory behavior and morphological variations.

## Materials and methods

### Rearing of FAW from Ghana

In July 2023, FAW larvae were collected from agricultural fields growing corn in the Soil and Irrigation Research Centre (SIREC) of the University of Ghana (6.132838°N, 0.074388°E), and the Akorley CHPS Compound (6.030209°N, 0.004689°E). These locations are near the town of Kpong in the eastern region of Ghana, situated in the Guinean forest–savannah ecoregion, which experiences a tropical climate with distinct wet and dry seasons. Much of the original vegetation has been replaced with agriculture, and corn is a frequent crop, which is often attacked by FAW outbreaks (Koffi *et al.*, 2020). These larvae were transported to the United Kingdom on July 26, 2023 under plant health authorization no. 120899/272309‐2. Larvae were reared in an artificial climate chamber at the Penryn Campus of the University of Exeter. Each larva was individually placed in a transparent plastic cup and fed artificial diet until pupation. Pupae were then transferred to new plastic cups with moist cotton. Adult moths were fed a 10% honey water solution. The climate chamber conditions were maintained at 25 ± 1 °C, 70% ± 10% relative humidity (RH), and 16 h L : 8 h D photoperiod. The larvae collected from Ghana mated and produced offspring (generation 1). We conducted our experiment using 2‐d‐old adults from generation 1 and the subsequent 2 generations (generations 2 and 3).

### Rearing of FAW from China

In March 2021, FAW larvae were collected from cornfields at the Yuanjiang Plant Protection Station, Yuanjiang County, Yuxi City, southern Yunnan, in Southwest China (23.601078°N, 101.978852°E) and reared in the laboratory. The larvae were stored in transparent plastic tubes and individually fed corn leaves. Pupae were transferred to plastic cups with moist cotton, and adult moths were fed a 10% honey water solution. The insects were kept in a cool indoor environment, avoiding direct sunlight, with natural ventilation and lighting to match local outdoor temperatures. Then, we directly measured the flight capability using 2‐d‐old adults.

### Flight capability testing

We used the same method to test the flight capabilities of FAW from China and FAW from Ghana. A rotational tethered flight mill system was used (Minter *et al.*, 2018), consisting of 8 independent mills allowing simultaneous measurement of 8 insects. Each mill had a steel arm suspended between 2 magnets, with the moth attached to one end, flying in a circular trajectory. Rotations were measured with a light detector to calculate flight parameters such as duration, distance, and average speed. This system has been used to assess the flight capabilities of various insects (Minter *et al.*, 2018; Jyothi *et al.*, 2021; Chen *et al.*, 2022). 2‐d‐old moths were cold‐anesthetized at −25 °C for 3 min to slow their movement, then their wings were spread and scales removed from the thorax. A 0.75 mm diameter, 2 cm long metal pin was attached to the thorax using cyanoacrylate glue, then inserted into a rubber tube on the steel arm of the flight mill. Flight tests were conducted at night for 10 h in darkness, maintaining optimal conditions (25 °C, 75% RH), excluding damaged adults.

### Morphological measurements

We measured morphological parameters for Ghanaian FAW. Pupal body mass was recorded, and after flight tests, the right forewings were removed with micro‐scissors. Wing images were taken with a camera and a spirit level, then imported into Photoshop to measure total length and area of the forewings. Aspect ratio and wing loading were calculated using standard formulas (Berwaerts *et al.*, 2002).

### Data analysis

Initial analysis of Ghanaian FAW flight data revealed significant differences in flight behavior among individuals, with some exhibiting very short maximum single flight duration (MSFD) and total flight duration (TFD). These individuals were classified as non‐migratory, while others were classified as migratory.

**Fig. 1:**
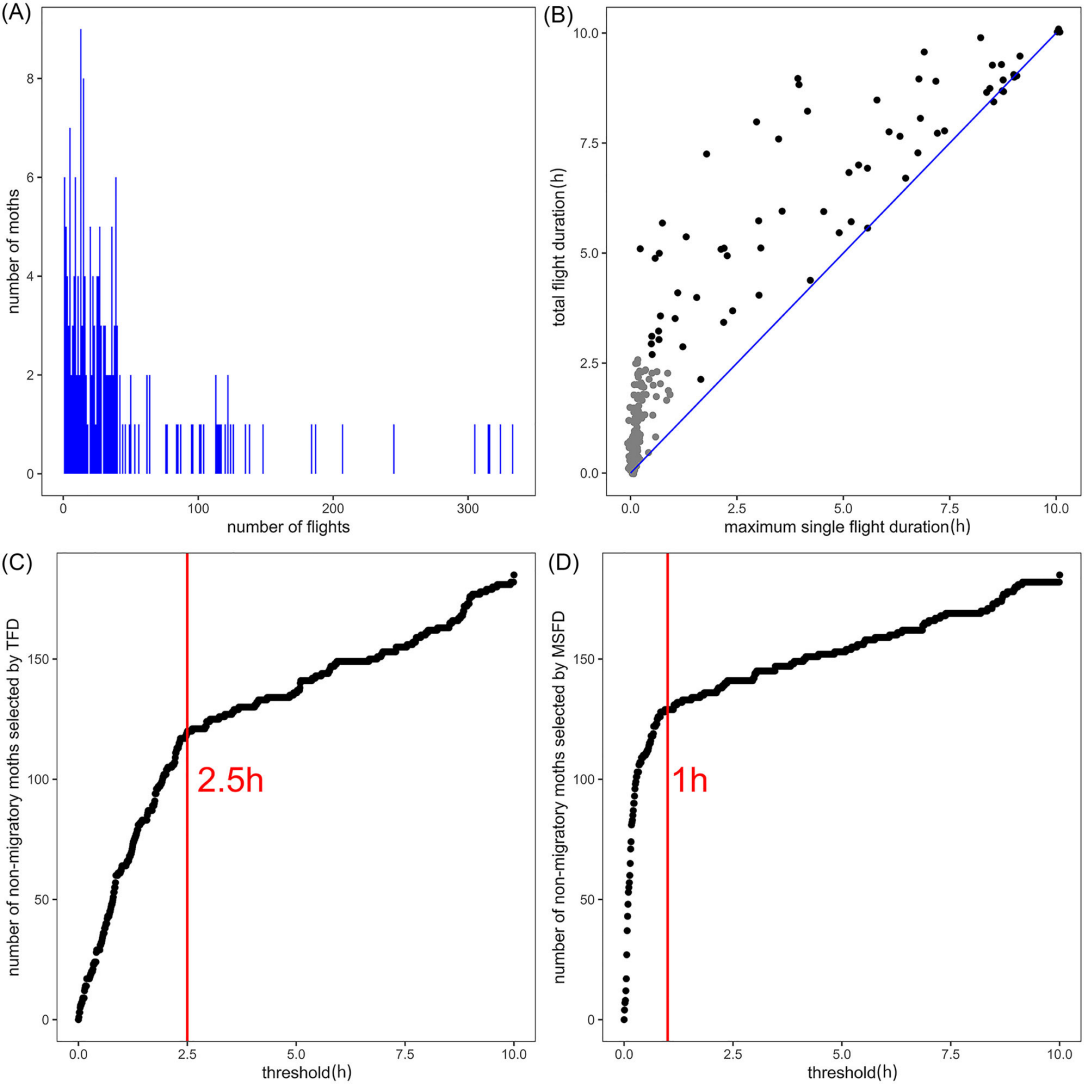
The flight patterns of all Ghanaian fall armyworms throughout the night. (A) The distribution of flight frequency per night. (B) Total flight duration (TFD) and maximum single flight duration (MSFD) of each moth. The experimental moths were divided into non‐migratory group (gray points) and migratory group (black points). We applied slight jitter to the points to prevent overlap. (C) The number of non‐migratory individuals under each threshold for TFD. (D) The number of non‐migratory individuals under each threshold for MSFD. Each red line marks the threshold at the inflection point, where a notable change in slope suggests clustering of individuals before this threshold in terms of flight duration. By considering the thresholds of 2 inflection points, we classified individuals into non‐migratory and migratory groups.

The specific classification steps are as follows. First, we generated 1 000 thresholds ranging from 0 to 10 h, with an interval of 0.01 h (36 s). Then, we calculated the number of individuals with MSFD and TFD below each threshold and represented this using scatter plots. The *x*‐axis represents different thresholds, and the *y*‐axis represents the number of individuals below the threshold (Fig. 1C, D). In both scatter plots, we observed a turning point where the slope before the turning point was significantly steeper than after it, indicating that many individuals' flight durations were clustered before the turning point. The turning point threshold for MSFD was approximately 1 h, while for TFD, it was approximately 2.5 h. Therefore, we defined FAW with a MSFD of less than 1 h and a TFD of less than 2.5 h as non‐migratory individuals, while the remaining individuals were defined as migratory individuals. Next, we compared the flight capability of FAW over 3 successive generations in Ghana.

When comparing the flight capability of FAW in Ghana and China, we minimized the impact of laboratory rearing by using only the flight mill data of the first‐generation FAW from Ghana (GH [lab 1st gen]) and field‐collected FAW from Yunnan, China (CN [field]). According to the above classification criteria, the FAW from Yunnan were also categorized into migratory and non‐migratory types.

Before analysis, we assessed data normality with the Shapiro–Wilk test and checked for homogeneity of variances with Levene's test. To evaluate differences in flight duration between groups, we used non‐parametric tests, including the Kruskal–Wallis test and the Wilcoxon test. To compare the proportion of non‐migratory and migratory individuals, we used the Chi‐square test. When analyzing the relationship between morphological characteristics and migratory behavior, we employed generalized linear models (GLM). The morphological variables considered included body mass, forewing length, forewing area, aspect ratio, and wing loading. First, we used binomial regression to analyze the morphological differences between migratory and non‐migratory FAW and named this regression model the morphology‐corrected flight ability classification (FAC model). Since the flight duration was constrained to 0−10 h, we transformed the TFD and MSFD into the percentage of time spent flying within 10 h. Then, we performed beta regression to examine the relationship between the morphological variables of migratory FAW and the percentage of flight duration. The model studying the relationship between morphological differences and the percentage of MSFD was named the morphology‐corrected percentage of MSFD of migratory moths (MSFD model). The model studying the relationship between morphological differences and the percentage of TFD was named the morphology‐corrected percentage of TFD of migratory moths (TFD model).

All the models mentioned above were constructed using the glmmTMB package, and the best models were selected using a stepwise regression approach to minimize the Akaike information criterion. Subsequently, we utilized the DHARMa package to diagnose our models and obtain the results (Brooks *et al.*, 2017; Hartig & Hartig, 2017; R Core Team, 2021). All data were analyzed in R (version 4.1.3, https://www.r‐project.org/, accessed on March 10, 2022).

## Results

### Flight capability of Ghanaian FAW

The experiment involved a total of 185 2‐d‐old *S. frugiperda* moths flown on the flight mill system (89 virgin females and 96 virgin males). We found that the majority of moths engaged in multiple flights with only 6 moths (3.2%) flying once during the entire night (Fig. 1A). As moths typically undergo a single prolonged flight in the field during migration, we considered the longest single flight was more representative of migration behavior. Consequently, in the subsequent sections, we separately analyzed the total flight duration (TFD) and the maximum single flight duration (MSFD) per night.

Many moths exhibited only short‐duration flights, which we interpreted as attempts to escape or other local flights rather than long‐range migratory flights (Fig. 1B). Based on variations in flight duration, we categorized the entire sample into migratory and non‐migratory groups. The non‐migratory group is defined by a MSFD of less than 1 h and a TFD of less than 2.5 h (Fig. 1B–D; detailed classification procedures are provided in the Materials and methods section). Among all individuals across all 3 generations, 119 moths were classified as non‐migratory (64.3%), and 66 moths were classified as migratory (35.7%). In the migratory group, 8 individuals demonstrated exceptional flight capabilities, with MSFD exceeding 8 h and TFD exceeding 9 h, among which 3 moths continuously flew for 10 h.

### Flight capabilities of Ghanaian FAW across 3 consecutive generations

**Fig. 2:**
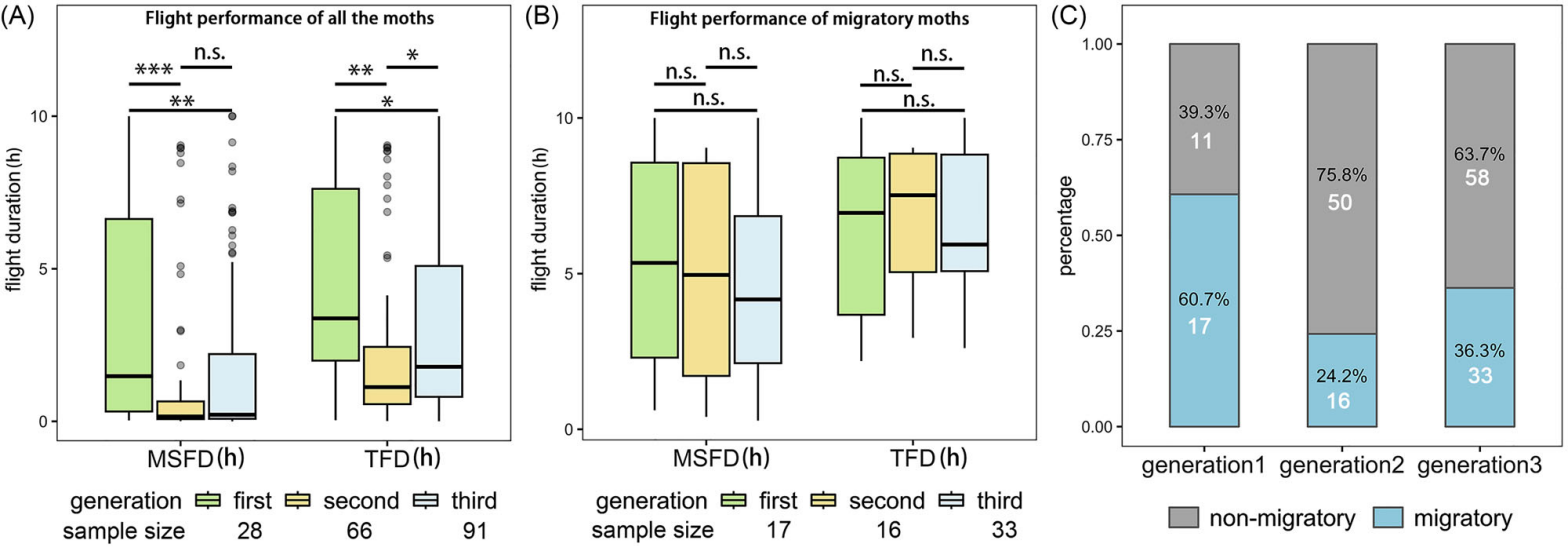
Comparative flight performance of Ghanaian fall armyworm across 3 consecutive generations. (A) The maximum single flight duration (MSFD) and total flight duration (TFD) of all samples across different generations. (B) The MSFD and TFD for migratory individuals across different generations. (C) The percentages of migratory and non‐migratory individuals across different generations, with white numbers indicating sample sizes. n.s., not significant, *P < 0.05, **P < 0.01, ***P < 0.001.

When considering all Ghanaian samples (including migratory and non‐migratory individuals), a significant decrease in flight duration was observed across 3 consecutive lab‐reared generations (Kruskal–Wallis tests; MSFD: *χ* ^2^ = 14.34, df = 2, *P* < 0.001; TFD: *χ* ^2^ = 111.89, df = 2, *P* = 0.003). This reduction occurred after only one generation of captive rearing (pairwise Wilcoxon tests; MFSD: *P* < 0.001; TFD: *P* = 0.003), but there was no further decrease between the 2nd and 3rd generations (pairwise Wilcoxon tests; MFSD: *P* = 0.29; slight increase in TFD: *P* = 0.03) (Fig. 2A). This intergenerational difference arose due to a reduction in the proportion of migratory individuals (Chi‐square tests; overall comparison: *P* = 0.003; generations 1 and 2: *P* = 0.002; generations 2 and 3: *P* = 0.15) (Fig. 2C), rather than a decrease in the flight duration of migratory individuals (Kruskal–Wallis tests; MSFD: *χ* ^2^ = 0.85, df = 2, *P* = 0.66; TFD: *χ* ^2^ = 0.21, df = 2, *P* = 0.90) (Fig. 2B).

### Comparison of the flight capabilities between Ghanaian and Chinese FAW

**Fig. 3:**
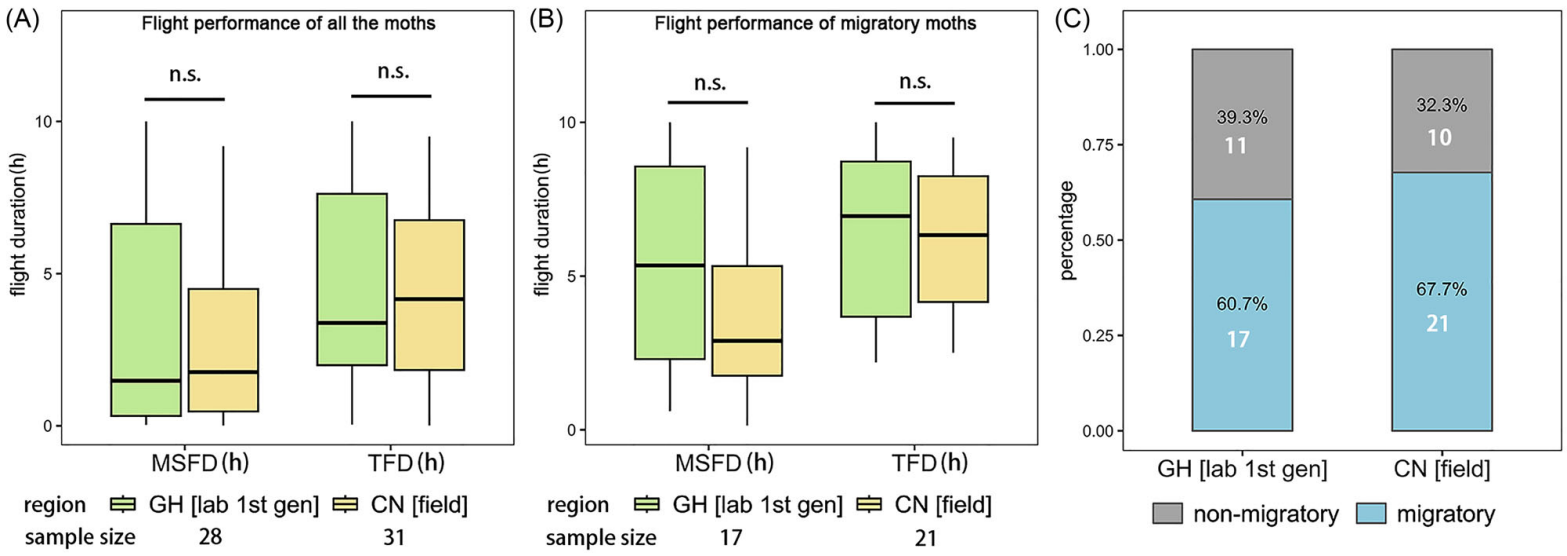
Comparative flight performance of Ghanaian fall armyworm (FAW) with Chinese FAW. (A) The maximum single flight duration (MSFD) and total flight duration (TFD) of the first generation of Ghanaian FAW (GH [lab 1st gen]) compared to Chinese wild FAW (CN [field]). (B) MSFD and TFD of GH [lab 1st gen] migratory individuals compared to CN [field] migratory individuals. (C) The percentages of migratory and non‐migratory individuals of GH [lab 1st gen] compared to CN [field], with white numbers indicating sample size. n.s., not significant.

To compare the flight capabilities between a field population of Chinese FAW and lab‐reared Ghanaian FAW, we extracted and compared the flight mill data of laboratory‐reared first‐generation Ghanaian FAW with that of wild Chinese FAW. There were no significant differences observed in terms of overall flight performance (Wilcoxon rank‐sum test; MSFD: *P* = 0.963; TFD: *P* = 0.9214) (Fig. 3A), proportion of migratory and non‐migratory individuals (Chi‐square test; *P* = 0.7713) (Fig. 3C), and flight performance of migratory individuals (Wilcoxon rank‐sum test; MSFD: *P* = 0.3785; TFD: *P* = 0.6811) (Fig. 3B).

### The relationship between flight capability and morphological features

**Fig. 4:**
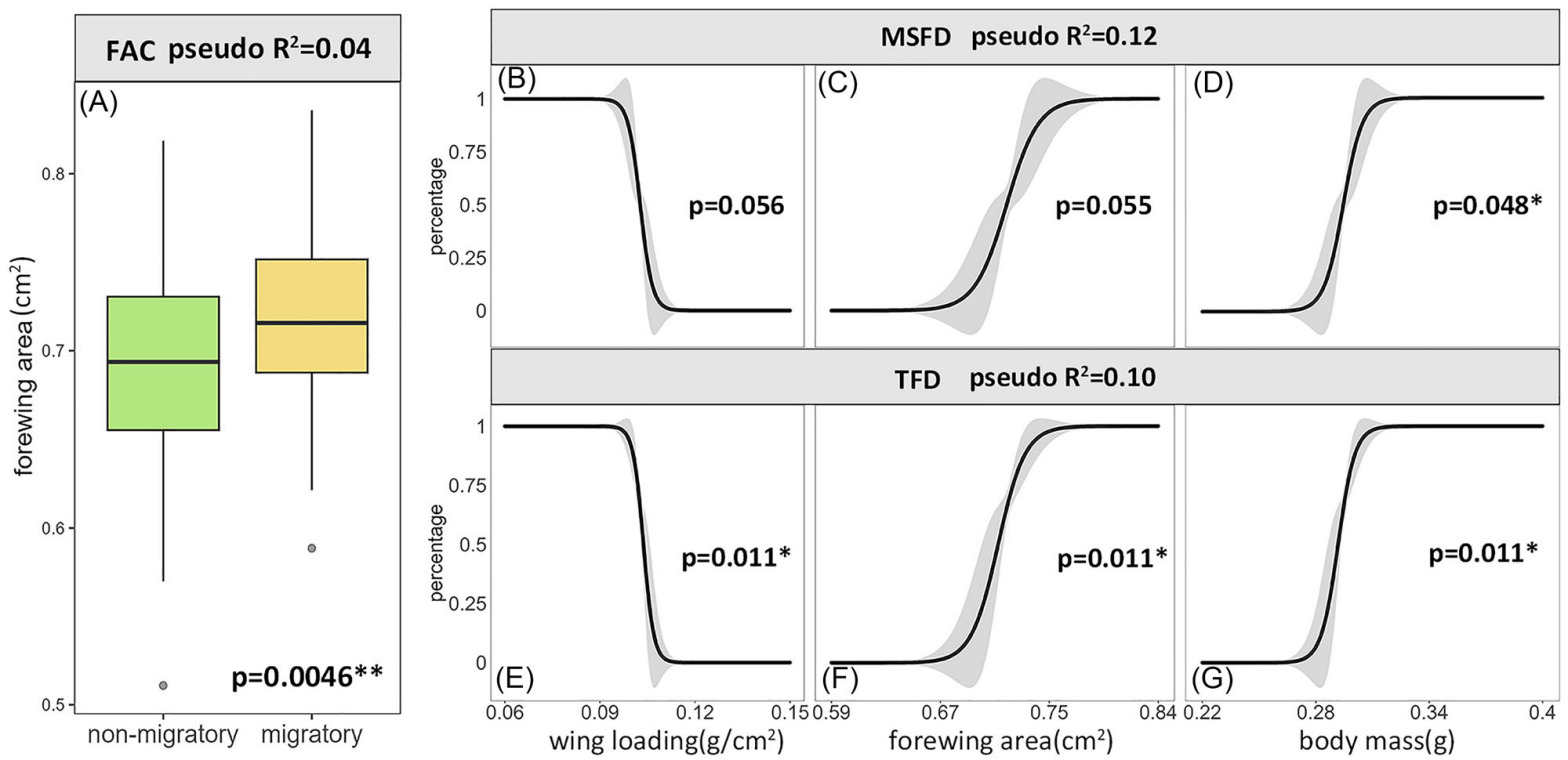
Visualization of the generalized linear models (GLM) constructed with morphological variables and the migratory performance of fall armyworm (FAW). The visualized models include the morphology‐corrected flight ability classification (FAC model), the morphology‐corrected percentage of the maximum single flight duration (MSFD) of migratory moths (MSFD model), and the morphology‐corrected percentage of the total flight duration (TFD) of migratory moths (TFD model). The relationship between all morphological variables included in the models (regardless of *P*‐value significance) and migratory performance is shown. (A) Comparison of forewing area between migratory and non‐migratory FAW in the FAC model. (B) Response curve of wing loading in the MSFD model. (C) Response curve of forewing area in the MSFD model. (D) Response curve of body mass in the MSFD model. (E) Response curve of wing loading in the TFD model. (F) Response curve of forewing area in the TFD model. (G) Response curve of body mass in the TFD model. The figure also represents the models’ pseudo‐*R* ^2^ values and the *P*‐values for the morphological variables. The detailed information of all models is shown in Table S1. *P < 0.05, **P < 0.01.

The flight performance of Ghanaian FAW is linked to 3 key morphological traits: body mass, forewing area, and wing loading. Moths with larger forewing areas are more likely to be migratory (GLM: *P* = 0.0046) (Fig. 4A, Table S1). In the migratory group, body mass is positively correlated with both MSFD and TFD (GLM; effect of body mass on MSFD: *P* = 0.048; effect of body mass on TFD: *P* = 0.011) (Fig. 4D, G, Table S1). Forewing area is positively correlated with TFD, while wing loading is negatively correlated with TFD (GLM; effect of forewing area on TFD: *P* = 0.011; effect of wing loading on TFD: *P* = 0.011) (Fig. 4E, F, Table S1). However, the explanatory power of morphological variables on flight performance is low (FAC model: pseudo *R* ^2^ = 0.04; MSFD model: pseudo *R* ^2^ = 0.12; TFD model: pseudo *R* ^2^ = 0.1) (Figs. 4 and S1, Table S1).

## Discussion

Although current research shows that FAW exhibits seasonal migratory behavior in the Americas and Asia (Westbrook *et al.*, 2016; Ma *et al.*, 2019; Chen *et al.*, 2020; Li *et al.*, 2020; Kenis *et al.*, 2022), its migration strategies in Africa remain unknown. In the tropical regions of West Africa, the minimal temperature variation throughout the year and the widespread potential annual habitats raises questions about whether West African FAW populations maintain the high migratory capability of the ancestral American populations. Our study provides evidence that the FAW in Ghana, West Africa, maintains a high migratory capability similar to that of a FAW population from Southwest China. However, this high migratory capability can rapidly disappear, as just 2 consecutive generations of laboratory rearing led to a significant decline in the proportion of migratory individuals among the Ghanaian FAW population. Additionally, we found that migrants have larger forewings, but the weak correlation between migratory ability and morphological traits suggests that genetic factors may play a more significant role in determining migration behavior.

In our study, the migration ability of the first‐generation FAW in Ghana shows no significant difference compared to a wild FAW population collected in Yunnan, Southwest China, indicating that FAW in Ghana maintain strong migratory capabilities, and providing evidence that FAW in West Africa continue to migrate. Why might migration be maintained in an environment with little temperature seasonality? One explanation is that the alternating dry and rainy seasons in West Africa, along with crop planting cycles, may intermittently reduce habitat suitability, prompting the FAW to migrate in search of edible crops such as corn and sorghum in neighboring areas (Niassy *et al.*, 2021; Guimapi *et al.*, 2022).

While Ghanaian FAW populations are clearly highly migratory, we do not know if they exhibit directional seasonal movements (i.e., northward in spring and southward in autumn) as seen in temperate environments (Chen *et al.*, 2023), or whether the movement directions will be more complex or even random. Unlike regions with significant temperature fluctuations, Ghana, near the equator, experiences minor temperature changes but distinct rainy and dry seasons. Rainfall patterns in West Africa are influenced by the annual movement of the Inter‐tropical Convergence Zone. Rainfall zones generally move from south to north during spring and summer, with its northernmost limit the southern fringe of the Sahara Desert, and then retract from north to south in autumn and winter (Efon *et al.*, 2023). Migrations of FAW may track the seasonal movements of the rainfall zones from wet tropical regions into the more arid regions to the north and back again, as has been shown for mosquitoes (Huestis *et al.*, 2019), a pattern that will be facilitated by the seasonally favorable prevailing winds. However, migratory activity, successful development and population growth will also be influenced by crop planting periods, which show more complex patterns than the simple seasonal change in rainfall and tracking these resources would lead to more complex patterns of migration involving movements along an east‐west axis as well as a north‐south axis (Niassy *et al.*, 2021; Guimapi *et al.*, 2022). Seasonal north‐south migration may not be an advantageous strategy to track the complex patterns of crop availability, as well as the risks associated with overshooting into the Sahara. It is thus quite possible that FAW populations in tropical regions like Ghana are more likely to engage in aseasonal, undirected migration in response to sudden environmental deterioration (Sappington, 2024). This type of migration does not have a specific spatial target and may simply serve to disperse offspring spatially, reducing the risk of high mortality if all offspring remain in deteriorating habitats (Sappington, 2024). Similar behaviors are observed in western corn rootworm (*Diabrotica virgifera virgifera*) and European corn borer (*Ostrinia nubilalis*), in which, despite their ability to undergo diapause for overwintering, some individuals still undertake undirected migrations (Sappington, 2024). To test this idea, the directional tendencies of FAW populations from tropical West Africa should be tested used virtual flight simulators that can ascertain seasonal preferences in flight direction (Dreyer *et al.*, 2018; Chen *et al.*, 2023).

Because FAW in West Africa maintains high migratory potential, eradication zones and neighboring uninvaded areas still face the risk of FAW invasion. This necessitates the development of comprehensive and sustainable pest management systems in West Africa. Additionally, methods predicting migration trajectories and potential distribution of FAW should be employed to assess the risk of West African FAW invasion to Europe (Early *et al.*, 2018; Li *et al.*, 2020; Huang *et al.*, 2022). The high proportion of migratory individuals in the first‐generation FAW in Ghana, with over 60% being migrants, supports the theory that migration allows individuals to mix over a larger area, maintaining gene flow among populations and ensuring the continuation of the migratory strategy. However, there is a large cover of natural forest separating West and East Africa, which may hinder gene flow between the FAW populations in these regions (Nagoshi *et al.*, 2022). Therefore, it is also essential to study the migration strategies of the FAW in East Africa.

After 2 generations of laboratory rearing, the proportion of migratory individuals in the Ghanaian FAW population significantly decreased. It may be because non‐migratory individuals have reproductive advantages. Insects need to balance energy allocation between migration and reproduction (Minter *et al.*, 2018). Many insects exhibit the “oogenesis‐flight” syndrome, where egg development and mating are suppressed during migration and only commence after it, whereas non‐migratory individuals can quickly reach sexual maturity and allocate resources to reproduction immediately after eclosion (Jiang *et al.*, 2011; Minter *et al.*, 2018; Wiklund & Friberg, 2022). Compared to migratory butterflies, their closely related non‐migratory congeners begin reproduction more quickly after eclosion (Wiklund & Friberg, 2022). Therefore, the migratory individuals of the Ghanaian FAW may have a later sexual maturity period compared to non‐migratory individuals. We began collecting eggs as soon as we observed signs of oviposition and stopped once we had collected enough eggs for breeding and experiments. It is likely that most migratory individuals only started laying eggs after we had finished collecting, resulting in the majority of the eggs we collected coming from non‐migratory individuals. Furthermore, rearing FAW in insect cages with limited space may cause non‐migratory individuals to exhibit lower activity levels and focus more on mating, while migratory individuals may expend energy attempting to escape, thereby missing mating opportunities. In the case of the codling moth (*Cydia pomonella*), the less active strain has higher reproductive capacity and longer lifespan than the more active strain, supporting our hypothesis (Gu *et al.*, 2006). These 2 factors might be crucial reasons for the higher proportion of non‐migratory individuals in the offspring. Due to reproductive competition, continuous laboratory rearing can significantly affect the flight capabilities of insects. Therefore, to obtain data that more accurately represent the migratory ability of wild populations, it is recommended to prioritize the use of wild populations or first‐generation laboratory‐reared individuals for measurements.

The laboratory conditions differ from those in the Ghanaian fields, which may lead to measurements that do not fully reflect FAW's migratory ability in the wild. Environmental cues like wind or temperature changes are crucial for initiating or sustaining insect migration (Chapman *et al.*, 2008; Chapman *et al.*, 2015; Dällenbach *et al.*, 2018). In the laboratory, FAW lacks these cues, which may affect its flight performance. We used a 16 h L: 8 h D photoperiod for FAW rearing to compare migration behavior with Chinese FAW, which differs from Ghana's natural photoperiod. Studies show that altering the photoperiod can change FAW's migratory orientation (Chen *et al.*, 2023), but it is unclear if it affects migratory duration.

In our Ghanaian FAW population, migratory individuals had larger forewing areas than non‐migratory ones. Many insects have stronger flight apparatus among migratory individuals, common in wing polymorphic species like the brown planthopper (*Nilaparvata lugens*) and milkweed bug (*Oncopeltus fasciatus*), where migratory individuals are long‐winged and non‐migratory individuals are short‐winged (Hegmann & Dingle, 1982; Lin *et al.*, 2018). In wing monomorphic insects, there are also a few reports, such as in migratory and non‐migratory monarch butterflies (*Danaus plexippus*), where migratory individuals have larger forewing areas, leading to more efficient flight (Freedman *et al.*, 2020). In Ghanaian FAW, body weight was positively correlated with migration ability. Larger moths might reduce water loss through a lower surface‐area‐to‐volume ratio and have ample flight fuel reserves, both advantageous for long‐distance migration (Roff & Fairbairn, 2007). However, all models constructed using morphological parameters and flight performance had poor goodness of fit, indicating that morphological differences are not the primary factors influencing FAW migratory performance. The evolution of morphological traits may be influenced by multiple behavioral demands, such as foraging, avoiding predators, and finding mates, in addition to migration (Freedman *et al.*, 2020). This might explain the weak explanatory power of wing morphology on migratory ability.

Environmental cues, genetic factors, and morphological characteristics all influence insect migration (Chapman *et al.*, 2008; Merlin *et al.*, 2009; Jones *et al.*, 2015; Jones *et al.*, 2016; Lin *et al.*, 2018; Minter *et al.*, 2018; Doyle *et al.*, 2022). In the current study, all individuals experienced the same environmental conditions, so differences in migratory performance are likely influenced mainly by genetic and morphological traits. The weak explanatory power of morphological characteristics suggests that genetic factors may play a decisive role in the migratory ability of FAW. In several insects, migratory activity has been proven to be heritable and in the monarch butterfly, flight muscle protein genes and compass orientation genes related to migratory behavior have been identified (Han & Gatehouse, 1993; Kent JR & Rankin, 2001; Merlin *et al.*, 2009; Zhan *et al.*, 2014; Dällenbach *et al.*, 2018). The molecular mechanisms underlying FAW migration behavior warrant further investigation to uncover the genetic and physiological foundations of this critical behavior.

## Disclosure

The authors declare no conflicts of interest associated with any aspect of this study.

## Supporting information




**Table S1** The association between morphological variables and migratory performance in the generalized linear models (GLM).

**Fig. S1** Scatter plots of Ghanaian migratory fall armyworm (FAW) flight performance against morphological parameters.
