# Supplementary material for: High migratory potential of fall armyworm in West Africa despite stable temperatures and widely available year‐round habitats
Source: Insect Sci. 2025 Jan 19;33(1):313–24. doi: 10.1111/1744-7917.13502 (PMC12905470; doi:10.1111/1744-7917.13502)
Supplement: Supplementary file 1 — Table S1 The association between morphological variables and migratory performance in the generalized linear models (GLM). [file INS-33-313-s001.docx]

**Supplemental Information**

**High migratory potential of fall armyworm in West Africa despite stable temperatures and widely available year-round habitats**

**Fan-Qi Gao, Hui Chen, Rosina Kyerematen, Gao Hu, Regan Early, Jason W. Chapman**

| Models’ name | Predictor Variables | Estimate±SE | P value | Pseudo R-square |
| --- | --- | --- | --- | --- |
| (A) FAC | (intercept) | -0.62±0.16 | 8.9e-05 *** | 0.04 |
|  | Forewing area | 0.48±0.17 | 0.0046 ** |  |
| (B) MSFD | (intercept) | -0.23±0.25 | 0.34 | 0.12 |
|  | Wing loading | -10.02±5.24 | 0.056 |  |
|  | Forewing area | 4.04±2.10 | 0.055 |  |
|  | Body mass | 7.02±3.55 | 0.048 * |  |
|  | Wing loading: Body mass | 0.25±0.21 | 0.22 |  |
|  | (intercept) | 0.36±0.22 | 0.099 | 0.10 |
|  | Wing loading | -11.73±4.64 | 0.011 * |  |
| (C) TFD | Forewing area | 4.75±1.86 | 0.011 * |  |
|  | Body mass | 8.00±3.15 | 0.011 * |  |
|  | Wing loading: Body mass | 0.42±0.19 | 0.032 * |  |

**Table S1** The association between morphological variables and migratory performance in the GLM models. (A) Morphology-corrected flight ability classification (FAC) (GLM with binomial distribution). (B) Morphology-corrected percentage of the maximum single flight duration (MSFD) of migratory moths (GLM with beta distribution). (C) Morphology-corrected percentage of the total flight duration (TFD) of migratory moths (GLM with beta distribution).


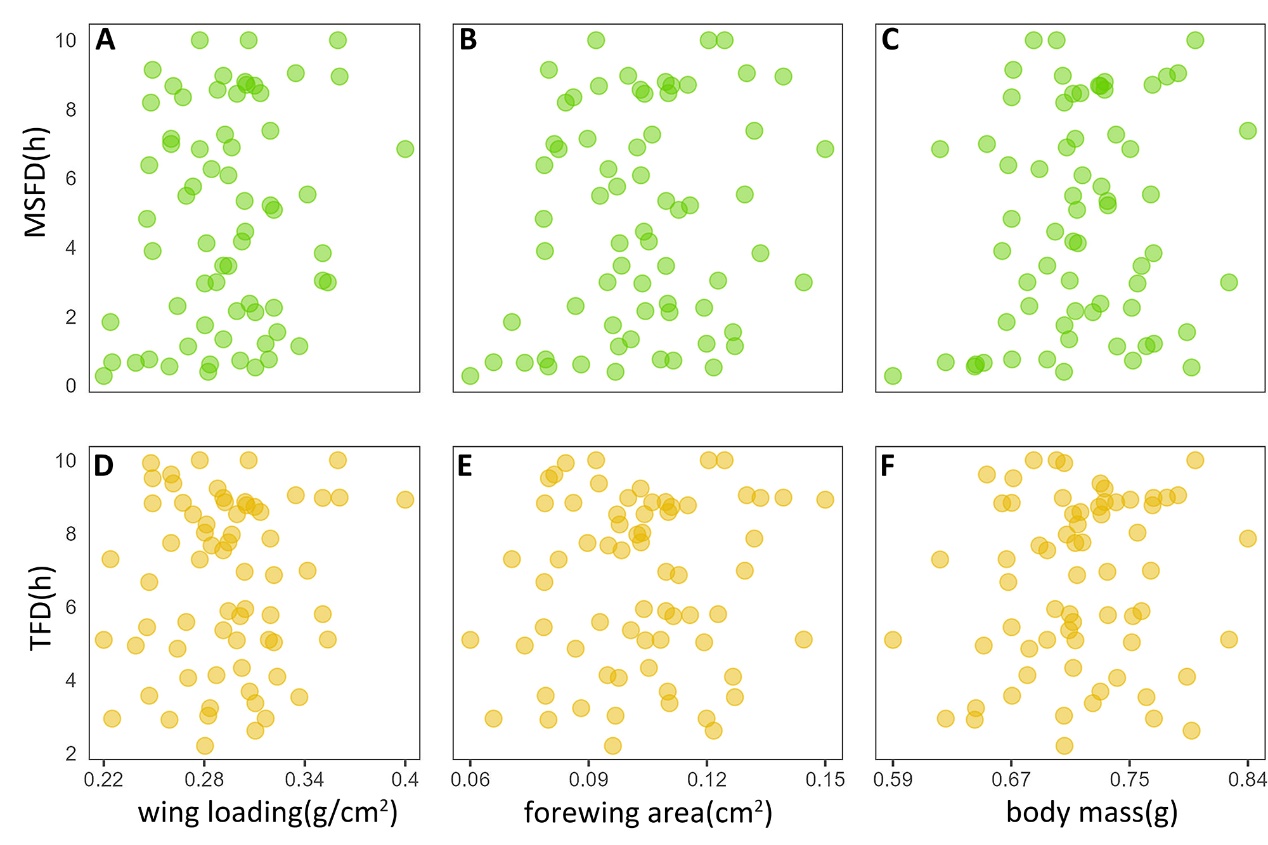


**Figure S1** Scatter plots of Ghanaian migratory FAW flight performance against morphological parameters. The relationships between three morphological parameters (wing loading, forewing area, and body mass) and flight durations (MSFD: maximum single flight duration; TFD: total flight duration) are shown. (A) The relationship between wing loading and MSFD. (C) The relationship between forewing area and MSFD. (D) The relationship between body mass and MSFD. (E) The relationship between wing loading and TFD. (F) The relationship between forewing area and TFD. (G) The relationship between body mass and TFD.
